# Commitment to Hypertension Control During the COVID-19 Pandemic: Million Hearts Initiative Exemplars

**DOI:** 10.5888/pcd19.210439

**Published:** 2022-08-04

**Authors:** Amena Abbas, Judy Hannan, Haley Stolp, Fátima Coronado, Laurence S. Sperling

**Affiliations:** 1 Division for Heart Disease and Stroke Prevention, National Center for Chronic Disease Prevention and Health Promotion, Centers for Disease Control and Prevention, Atlanta, Georgia; 2 Oak Ridge Institute for Science and Education, Oak Ridge, Tennessee; 3 ASRT Inc., Atlanta, Georgia; 4 Department of Medicine, Division of Cardiology, Emory University School of Medicine, Atlanta, Georgia

## Abstract

Hypertension is a major risk factor for cardiovascular diseases, but 3 of 4 US adults do not have their blood pressure adequately controlled. Million Hearts (US Department of Health and Human Services) is a national initiative that promotes a set of priorities and interventions to optimize delivery of evidence-based strategies to manage cardiovascular disease, including hypertension. The COVID-19 pandemic, however, has disrupted routine care and preventive service delivery. We identified examples of clinical and health organizations that adapted services and care processes to continue a focus on monitoring and controlling hypertension during the pandemic. Eight Hypertension Control Exemplars were identified and interviewed. They reported various adapted care strategies including telemedicine, engaging patients in self-measured blood pressure monitoring, adapting or implementing medication management services, activating partnerships to respond to patient needs or expand services, and implementing unique patient outreach approaches. Documenting these hypertension control strategies can help increase adoption of adaptive approaches during public health emergencies and routine care.

**Summary**

**What is already known on this topic?**

The COVID-19 pandemic has caused unprecedented disruptions in routine care and chronic disease management. As hypertension is the most common modifiable risk factor for cardiovascular events, it is imperative that, even during disruptions in care, hypertension control remains a priority.

**What is added by this report?**

In response to the challenges presented by COVID-19, clinicians and health care organizations implemented various and unique strategies to respond to patient needs and expand services to monitor hypertension, demonstrating that even during a time of public health crisis, focus on improving hypertension control is possible.

**What are the implications for public health practice?**

The findings highlight how health care and public health programs have been able to accelerate innovation and adapt services for continuity of care and hypertension control. This may help inform future efforts to improve health care delivery related to hypertension control, during and after a public health emergency.

## Introduction

Hypertension is a major risk factor for several chronic diseases, including stroke, heart disease, and other CVDs (1). Blood pressure consistently at or above 130/80 mm Hg is considered hypertensive (1). It is also a significant primary or contributary cause of death in the US (2). Furthermore, estimates indicate that approximately 50% to 75% of adults with hypertension do not have their blood pressure adequately controlled (3).

Infections with SARS-CoV-2, the virus that causes COVID-19, has resulted in approximately 900,000 deaths to date and has had an enormous effect on health and health care in the US (4). In March 2020, shelter-in-place orders went into effect, and states declared a state of emergency as a result of considerable community transmission of COVID-19 (5). Disruptions in routine and nonemergent medical care were reported with substantial decreases in patient visits and restricted hours of operation (6). An estimated 41% of US adults initially avoided or delayed medical care because of COVID-19 concerns or were encouraged to postpone routine appointments with their health care team if determined to be at high risk for COVID-19 (7). Underlying serious health conditions, including hypertension, possibly increase the likelihood of severe COVID-19–related illness (8). Thus, it is imperative that, even during disruptions in care, hypertension control remains a priority.

## Purpose and Objectives

Million Hearts (MH), a national initiative to prevent 1 million heart attacks, strokes, and other cardiovascular events within 5 years, focuses the attention of public health and health care partners on a small set of priorities to optimize delivery of evidence-based strategies to achieve specific targets in aspirin use, blood pressure control, cholesterol management, and smoking cessation. Since 2012, the MH Hypertension Control Challenge has recognized clinicians, care practices, and health systems that achieve blood pressure control rates of 70% through 2017 and 80% control through 2019 as Champions (9). The COVID-19 pandemic, however, has disrupted clinical care and preventive service delivery, altered quality improvement support programs, and stalled many public health program activities and initiatives (10). In response to the COVID-19 pandemic, and because of the potential risk of infection during in-person visits, federal agencies relaxed telemedicine regulations and increased funding to support its implementation, which resulted in a substantial increase in the utilization of telemedicine (11). However, the use of patient-generated blood pressure measurements is not uniformly captured in the medical record or universally accepted for reporting data to certain blood pressure control clinical quality measures. Despite these disruptions in care and reporting practices, MH remained committed to recognizing those who continued to address hypertension control. This report identifies and describes lessons learned from clinicians and health care organizations that adapted routine practice or care to maintain a focus on hypertension control during the COVID-19 pandemic.

## Evaluation Methods

MH queried its public and private sector partners to identify Hypertension Control Exemplars. Selection of Exemplars was based on the following criteria: 1) clinicians, medical centers, or health system support organizations that altered patient care or services or implemented new approaches in response to challenges presented by COVID-19 to prioritize hypertension control, 2) uniqueness of intervention, 3) community improvement individuals and organizations that served or prioritized under-resourced or patient populations who were disproportionately affected by COVID-19 or at risk for uncontrolled hypertension, and 4) community improvement individuals and organizations that demonstrated or documented qualitative or quantitative results of their hypertension control efforts and strategies (eg, percentage of patients with hypertension under control, number of patients reached, outcomes of implemented strategies, other benefits measured). A goal was to identify Exemplars across varied settings, including those delivering clinical care directly and organizations supporting health systems.

In addition to identifying eligible Exemplars, MH staff conducted virtual interviews by using a structured questionnaire to gather qualitative and quantitative data related to hypertension control efforts. The questionnaire gathered information on 1) general demographics and clinical information on the overall patient population and information specific to hypertensive patients, 2) adaptations to routine patient care or health services to monitor and control hypertension, and 3) outcomes and successes of hypertension control strategies, as well as challenges or barriers encountered during implementation. Interviews were audio recorded and transcribed. The Centers for Disease Control and Prevention (CDC) reviewed this study for human subjects’ protection and determined it to be nonresearch.

## Results

A total of 8 Hypertension Control Exemplars were identified (Box). Four Exemplars were clinical practices (California Right Meds Collaborative, Community Health & Wellness Partners, Jessie Trice Community Health Center, and Philadelphia FIGHT), including 3 federally qualified health centers that provided direct patient care. Four were health system support organizations (Aledade, Inc, Missouri Hospital Association, Quality Insights, Inc, and YMCA of Central New York). Exemplars providing clinical services served a median population of 7,315 patients, mostly in geographically urban settings (urban service areas). Overall patient characteristics include a 58% racial and ethnic minority mean, 6% with English as second language, 32% with Medicaid coverage, and 26% uninsured. Among Exemplar clinical practices, the mean percentage of patients with a diagnosis of hypertension in 2020 was 31%. The mean blood pressure control rate reported was 65% in 2019, 60% in 2020, and 61% in July 2021. Clinical practices reported a decrease in blood pressure control rates between 2019 and 2020, but an increase or no change between reported rates in 2020 and 2021.

**Box. Million Hearts Hypertension Exemplars and Patient Population Characteristics of Clinical Practices, 2021**

**Practice or medical center**

California Right Meds Collaborative
Community Health & Wellness Partners
Jessie Trice Community Health Center
Philadelphia FIGHT

**Health system support organizations**

Aledade Inc.
Missouri Hospital Association
Quality Insight Inc
YMCA of Central New York

**Patient characteristics (Some respondents reported multiple characteristics for patient population. Percentages do not total 100%.)**

Source is HRSA (Health Resources and Services Administration) UDS (Uniform Data System) Database.

**Number of patients** (mean, SD) — 15,000 (18,374.1).

**Racial or Ethnic minority — **57.9%. Minority status of patients was determined by Exemplars.

**English as second language — **6.0%

**Enrolled in Medicaid —** 32.1%

**Uninsured** — 25.9%

**Percent with hypertension (mean, SD)** — 30.8% (15.3)

**Hypertension control rates during data collection (mean, SD)**

2019 — 65.0% (9.4%)
2020 — 60.3% (8.1%)
2021 — 60.8% (6.9%) is most recent rate available to report in calendar year 2021, June–August

We detailed strategies and reported outcomes for each Exemplar (Table). The average percentage of patients with hypertension that Exemplars reported reaching through their various strategies and interventions was 45%. Exemplars also reported implementing hypertension control strategies that focused on specific characteristics or demographics of patients with hypertension. Exemplars providing clinical services reported using telehealth services, adapting self-measured blood pressure monitoring, establishing drive-thru or parking lot clinics to measure blood pressure, developing medication management strategies, partnering with community organizations, and creating strategies for patient outreach including using population health software to develop high-risk registries for outreach. Several Exemplars reported direct outreach to patients through methods such as delivery of prescription medications to those with comorbidities including hypertension, or phone calls to follow up and assess patient health.

**Table:** Strategies and Outcomes by Hypertension Control Exemplars in Implementing Hypertension Control Strategies in Response to COVID-19

Exemplar	Modifications to routine practice or care for blood pressure control during COVID-19	Reported outcomes
**Clinical practice or health center**		
California Right Meds Collaborative	• Piloted program to utilize community pharmacy network to deliver comprehensive medication management services • Trained pharmacists to provide video and phone telehealth services • Developed registry for outreach to high-risk beneficiaries of participating health plans • Facilitated access to home blood pressure monitors, either through health plan or provided by pharmacy	• Reduced systolic blood pressure of hypertensive patients by 23 mm Hg • Achieved blood pressure control rate of more than 85% in previously uncontrolled hypertensive patients
Community Health & Wellness Partners	• Program care coordinator messaged patients twice a week to check in on health of patients and promote awareness of telehealth services • High-risk patients enrolled by telephone into their chronic care management program • Used onsite parking lot to serve patients • Hypertensive enrollees received text messages with information about DASH and blood pressure management • Clinical pharmacist used telehealth video feed to cover other practice locations and increase reach to patients	• Converted approximately 12% of cold messages from a nurse into active patients • 97% of patients respond to a message • In evaluating service, 91% of patients rated the new adapted services at least a 9 out of 10
Jessie Trice Community Health Center	• Established a drive-through clinic where chronic care patients could get their blood pressure measured (and hemoglobin A1c tested) • Self-monitored blood pressure platform that used cellular data to remotely monitor chronic care patients • Used readings and data transmitted back to adjust the patient’s medication or request an in-person visit to the office if needed • Provided virtual meetings and classes with behavioral health, nutritionists, and medicine management specialists to speak to patients	• 900 patients received home blood pressure measurement device, 1,055 blood pressure screenings • Enrolled 72 in remote monitoring through Bluetooth and 110 self-monitored; reported measures during follow-up (onsite or telehealth visit)
Philadelphia FIGHT	• Established hotline to health centers to have live health care available to patients • Delivered medications to patients unable to receive at-home delivery through pharmacy • Identified high risk patients with chronic conditions for direct outreach • Provided phone instruction to patients on how to accurately use at-home blood pressure measurement devices	• Reached more than 5,000 patients • Delivered medication to 600 patients • Provided medication management support to 1,200 patients
**Health system support or community health organization**		
Aledade Inc	Transformed AMA MAP program to virtual pilot • Created dashboards to monitor trends in blood pressure control and dashboard with the list of uncontrolled patients • Distributed blood pressure kits to expand self-monitored blood pressure services • Collected blood pressure monitoring data from participating clinics - disseminated results to increase awareness and outreach to populations at increased risk for uncontrolled hypertension • Trained staff at pilot sites on how to bill for telehealth services	• Successfully scaled pilot to 3 sites covering approximately 7,000 patients • Improved blood pressure control rate from 73% to 82% within 6 months • Expanded program in virtual format to remaining sites (total 30)
Missouri Hospital Association	• Provided pregnant and postpartum women at highest risk for a hypertension-related complication a home blood pressure measurement device • Worked with established programs to get blood pressure devices to a network of hospitals and clinicians • Focus support on cardiovascular complications related to pregnancy • Provided educational materials on hypertension management and amplified telehealth for hypertension treatment	• 3,000 blood pressure cuffs distributed to 35 sites in Missouri
Quality Insights, Inc.	• Adapted quality improvement approach to continue supporting network of practices during COVID • Assisted participating clinics to adjust to telehealth and modified workflows • Provided technical assistance to participating health care organizations by shifting site visits to virtual visits and meetings • Assisted clinics in accessing blood pressure kits by providing resources and guidance in working with insurance companies • Partnered with pharmacists to work with physicians on medication management therapy	• Supported participating practices to achieve blood pressure control rate of 83% • Worked with 50 pharmacists for medication management services
YMCA of Central New York	• Established 16-week employer-based hypertension control and lifestyle modification program • Assisted and coached in use of home blood pressure monitors and lifestyle modification through virtual one-to-one office hours • Discussed blood pressure readings during office hours (eg, stressors, adherence to diets, exercise) • Offered virtual classes and peer support to participants	• Virtual format increased participant engagement • Participants reported high satisfaction with program

Abbreviations: AMA MAP, American Medical Association Measure Accurately, Act Rapidly, Partner With Patients; blood pressure, blood pressure; DASH, Dietary Approaches to Stop Hypertension; self-measured blood pressure monitoring.

Health system support organizations reported several approaches to respond to critical needs of clinical practices. Approaches included distributing home blood pressure measurement devices, adapting existing program activities to be delivered by using virtual platforms, and leveraging existing partnerships and innovative payment models to bolster and sustain hypertension activities. The organizations also worked to bolster remote blood pressure monitoring by providing resources to remove barriers or ease bureaucratic challenges for clinical practices to immediately access blood pressure monitoring devices for their patients. Others led efforts to focus on vulnerable patient populations, such as 1 Exemplar that leveraged statewide partnerships to focus on hypertension control among pregnant and postpartum women at highest risk for hypertension-related complications. Health system support organizations also provided resources to identify patients who were at increased risk for adverse cardiovascular events by encouraging participating clinical practices to use dashboards and other analytical software to target patients and monitor trends in blood pressure control.

Several Exemplars reported improved hypertension control rates resulting from supportive practice networks. Examples include a quality improvement network achieving blood pressure control rates of 83%, a medication therapy management pilot achieving a blood pressure control rate of over 85% in previously uncontrolled hypertensive patients, and a pilot delivered in a virtual format that improved control rates from 73% to 82% in its clinical sites in 6 months. Other highlighted outcomes include patient engagement resulting in positive feedback, results from expanded outreach efforts such as medication delivery to more than 600 patients, and blood pressure measurement device distribution. Collectively, Exemplars were successful in distributing over 4,000 devices for self-monitored blood pressure monitoring.

Exemplars reported several challenges and barriers to hypertension control, including limited available funds to meet the demand for blood pressure measurement devices for self-monitored blood pressure monitoring and in bringing public insurance programs to provide blood pressure cuffs to patients. Furthermore, although telehealth services were expanded considerably during the pandemic, many patients were unfamiliar with the technology or had limited access to high-speed internet for stable virtual visits. An ongoing need also exists for flexibility to better streamline processes and workflows to ensure smooth transitions in adapting services throughout the pandemic.

## Implications for Public Health

Our report summarizes hypertension control strategies that MH Exemplars implemented in response to the disruptions to routine medical care during the COVID-19 pandemic. Data support the fact that frequent interactions with clinical staff are essential to chronic disease management and during temporary disruptions in access to health care for hypertensive patients when a natural disaster results in increased rates of uncontrolled hypertension (12). Identifying innovative strategies and sharing lessons learned from Exemplars might help inform future efforts to improve health care delivery related to hypertension control during and after a public health or environmental emergency.

Patients with existing medical conditions have experienced poor outcomes in the setting of an emergency, including difficulty accessing emergency services and routine care (13). People living with chronic diseases, including hypertension, are at an increased risk of adverse health outcomes in the face of public health emergencies, and this risk increases exponentially with a prolonged crisis (12). Many communities are not adequately prepared to meet the needs of people living with chronic diseases during a public health emergency. MH Exemplars have demonstrated resilience and tenacity in their mission to control hypertension by accelerating innovation and adaptation of their services, despite many challenges through strategies that may have otherwise taken years to integrate into the services and workflow of these clinics and organizations.

Disruptions in access to care as a result of the pandemic have exposed the need to have a more integrated health system with potentially expanded roles for care team members such as community pharmacists. For example, an Exemplar implemented an accelerated pilot program focused on comprehensive medication management using a network of community pharmacists, physicians, and health plans. Studies have demonstrated that pharmacy-delivered medication therapy management can improve health outcomes for hypertensive patients and those with other chronic conditions or comorbidities (14). The medication therapy management program drove collaboration between community pharmacists and primary care physicians, resulting in hypertension control rates of more than 85%.

Many health care organizations and primary care practices used new and adapted existing resources to rapidly move to virtual care. Emergency funds provided by the passage of the CARES (Coronavirus Aid, Relief, and Economic Security) Act were allocated for “provider relief . . . related to expenses or lost revenues that are attributable to coronavirus” (15). Several Exemplars leveraged these emergency funds to immediately respond to the need of their patients and support expenses related to telehealth services, and to provide blood pressure measurement devices, other educational materials, and software for patient care. Moreover, Exemplars demonstrated that existing partnerships facilitated rapid implementation of their interventions and supported ongoing efforts. This activation of a ready network of partners contributed to a rapid response to gaps in care related to COVID-19 for health services and access.

The study is subject to limitations. First, data and outcomes were self-reported. Collecting data on patient outcomes or evaluating changes in blood pressure control rates as a result of the strategies implemented might have been useful. Second, a small number of Exemplars reported strategies focused on specific patient demographics; therefore, we were not able to examine or explicitly address the impact on health disparities. As there are disparities in hypertension control as well as COVID-19 infection and outcomes, it is crucial to document successful strategies for populations at higher risk. Lastly, as the data were obtained from a sample of a small number of clinics and organizations, the results and outcomes are not generalizable to the broader population of hypertension control program partners.

The COVID-19 pandemic has presented many challenges to hypertension control, including unprecedented disruptions to routine care and chronic disease management. The small-scale implementation of comprehensive interventions during a public health crisis allowed Exemplars to demonstrate promising results and sustainable impacts, captured the interest of relevant community members or organizations, and encouraged decision makers and partners to adopt and scale intervention models to their respective health systems. The examples presented demonstrate that even during a time of crisis, focusing on and achieving hypertension control is possible. Many of the adaptations made by these Exemplars can and will continue during noncrises and add important insights into creative solutions to long-standing problems, such as improving hypertension control.
